# Trichinellosis knowledge and preventive practices in Mapuche communities of southern Chile: Evidence for targeted One Health implementation

**DOI:** 10.1016/j.onehlt.2026.101366

**Published:** 2026-02-17

**Authors:** Tania Grant-Riquelme, Yanina Poblete, Marcela Fresno, Cecilia Baumberger, Italo Fernandez Fonseca, Christopher Hamilton-West, Francisca Di Pillo

**Affiliations:** a Universidad Católica de la Santísima Concepción, Grupo Consolidado de Innovación en Promoción de la Salud y Prevención de la Enfermedad (PROSALUD), Facultad de Medicina, Departamento de Ciencias Básicas y Morfología, Chile; b Trabajo Final de Grado Magíster One Health – Una Salud, Universidad de Las Américas, Santiago, Chile; c Núcleo de Investigación en One Health (NIOH). Facultad de Medicina Veterinaria y Agronomía, Universidad de Las Américas, Campus Providencia, Manuel Montt 948, Santiago, Chile; d Departamento de Medicina Preventiva, Facultad de Ciencias Veterinarias y Pecuarias, Universidad de Chile, Santiago, Chile; e Departamento de Microbiología, Facultad de Ciencias Biológicas, Universidad de Concepción, Concepción, Chile

**Keywords:** Trichinellosis, One health, Indigenous health, KAP survey, Chile, Mapuche

## Abstract

Trichinellosis is a foodborne zoonosis that persists in rural areas where backyard pig farming and informal slaughtering occur. In Indigenous communities, prevention depends on interconnected human behaviors, animal management, and local environmental factors. This study aimed to describe knowledge and preventive practices regarding trichinellosis among Mapuche communities in south-central Chile and to assess whether higher knowledge is associated with safer practices, while controlling for community and demographic factors.

A cross-sectional survey was conducted among 180 adults from nine Mapuche communities in Contulmo, Chile. Knowledge and preventive practices were summarized using a score ranging from 0 to 9. The association between knowledge and practices was analyzed with adjusted regression models controlling for community, sex, and age group. Principal component analysis identified common patterns among items. Results indicated that knowledge was generally high (average 7.47/9), whereas preventive practices were lower (average 6.21/9), with gaps in routine hygiene and in the disposal of infected meat. Preventive practices improved with increased knowledge after adjustments (β = 0.362 for each additional correct knowledge item; 95% CI 0.208–0.516; *p* < 0.001). Some community differences in practices persisted even after demographic adjustments. PCA revealed two main dimensions related to prevention behaviors and food-preparation misconceptions. The conclusions indicate that higher levels of knowledge correlate with safer practices, yet implementation gaps and community differences persist. One Health strategies should integrate culturally appropriate risk communication with community-specific support measures to enhance the practicality of testing and safe carcass disposal.

## 1 Introduction

Trichinellosis is a foodborne parasitic zoonosis [1] acquired mainly through consumption of raw or undercooked meat containing viable *Trichinella* larvae, most often from domestic pigs and occasionally from wild mammals [2], [3]. Prevention depends on intersectoral actions across the food chain, from pig production and slaughter to household meat preparation [4], including safe slaughter practices, veterinary inspection or testing when available, and thorough cooking [5]. In rural areas where households raise pigs, and informal slaughter occurs [6], exposures may persist despite surveillance and control efforts, making trichinellosis a useful model for understanding how human behaviors, animal management, and local environments jointly shape risk [7].

From a One Health perspective, the risk of trichinellosis originates at the human–pig–environment interface, where prevention relies on coordinated actions at multiple levels (households, animal management, and community settings) [8]. On the human side, decisions about slaughter, meat handling, and cooking determine whether contaminated meat is eaten, while access to timely diagnosis and healthcare affects detection and outbreak response. On the animal side, husbandry practices that increase pigs' exposure to infected tissues—such as poor biosecurity, rodent access to feed, and unsafe disposal of carcasses or offal—can maintain transmission within domestic cycles and may link domestic risks to surrounding wildlife and synanthropic hosts [6], [9]. Environmentally, waste management and rodent ecology can further facilitate disease maintenance and spread, especially where humans, domestic animals, and wildlife share spaces and resources [10], and where carcass remains are accessible [9], [11], [12]. In Indigenous communities, these pathways are additionally shaped by livelihoods, local governance, and stakeholder engagement in interventions that are co-designed and culturally safe—key themes in One Health work with Indigenous populations [13], [14], [15], [16].

Empirical evidence indicates that misconceptions about meat safety and parasite inactivation are common in rural Chile, and that higher levels of knowledge do not necessarily lead to consistent preventive practices [17], [18]. However, few studies have measured how trichinellosis-related knowledge and practices differ among Indigenous communities within the same territory or examined the knowledge-practice relationship while considering key social strata. Additionally, KAP (Knowledge, Attitude, and Practice) instruments generate multivariate response patterns that are more useful for intervention design when summarized into interpretable behavioral dimensions, rather than treated as isolated questions.

Accordingly, the objective of this study was to assess trichinellosis-related knowledge and preventive practices and quantify their association, while identifying co-occurring behavioral patterns to inform One Health prevention strategies in Mapuche communities in Contulmo, Chile.

## 2 Materials and methods

### 2.1 Study area, design, and community engagement

This cross-sectional study was carried out in the commune of Contulmo (Arauco Province, Bío Bío Region, south-central Chile), a predominantly rural area within the Nahuelbuta Range. The target population included adults (aged 18 and older) who identified as Mapuche and lived in Mapuche communities within the commune.

An official list of Mapuche communities in Contulmo, including their names and locations, was obtained from the Municipality of Contulmo through a formal transparency request. Initial engagement was coordinated through the Municipal Indigenous Affairs Office, which held an informational meeting with community leaders to introduce the research team, outline the study objectives, and explain the informed consent and questionnaire procedures. All 18 Mapuche communities in the area were invited to participate, and nine of them agreed and were included in the study. For reporting purposes, communities are numbered from 1 to 9.

### 2.2 Sampling strategy and participant recruitment

Community participation was non-probabilistic and based on convenience, determined by each community's willingness to participate and the feasibility of implementation within the study period. This approach was selected because research access in Indigenous territories requires community authorization and culturally appropriate engagement processes; therefore, participation necessarily depended on community-level agreement and local governance structures. Participating communities tended to be those with stronger communication and coordination with municipal services, which may limit representativeness at the commune level.

Within participating communities, recruitment also followed a convenience approach. After ethical approval, printed informed consent forms and questionnaires were delivered to the Municipal Indigenous Affairs Office and then handed out to community leaders during a follow-up coordination meeting. Community leaders offered the study materials to Mapuche adults in their communities who were willing to participate. Participants completed the questionnaire independently (self-administered, self-report) and returned the materials to community leaders, who then sent them back to the Municipal Indigenous Affairs Office for transfer to the research team. This approach was chosen to reduce barriers to participation and to respect local preferences expressed by community leaders, including concerns about time constraints and discomfort associated with external interviewers circulating in Indigenous territories during a sensitive local socio-political period [19], [20], [21]. Since distribution and return were managed through community channels rather than a controlled sampling frame, individual-level refusal rates could not be reliably measured. Using community gatekeepers may also introduce selection bias (e.g., through uneven outreach to certain households) and social desirability bias in responses. These limitations are taken into account when interpreting the results.

### 2.3 Questionnaire instrument and scoring

A structured questionnaire was developed based on an existing knowledge–attitudes–practices (KAP) instrument previously used in Chile [17] and adapted to the local context. Content validity was evaluated through expert review by six professionals, resulting in a final instrument of 18 items divided into two dimensions: Knowledge (9 items) and Preventive practices (9 items). The Knowledge dimension assessed awareness of key concepts in trichinellosis (e.g., affected hosts, cause, transmission routes, clinical signs, high-risk meats, and misconceptions about inactivation). The Preventive practices dimension evaluated behaviors related to prevention and response (e.g., cooking practices, testing home-slaughtered pork, disposal of infected meat, and health-seeking actions). All items were scored as correct or incorrect according to predefined rules: 1 for responses deemed correct or expected under established trichinellosis prevention guidelines, and 0 for all others. Two raw dimension scores were calculated for each respondent.

• Knowledge sum score: 0–9
• Practices sum score: 0–9

The internal consistency of the dichotomous instrument was assessed using the Kuder–Richardson Formula 20 (KR-20), which produced an overall coefficient of 0.71, indicating acceptable reliability for group comparisons.

Data were collected from June to August 2023 using paper-based questionnaires in a self-report format. Participants reviewed and signed the informed consent form before completing the questionnaire. Completed materials were returned through the community distribution pathway described above and entered into the study database by the research team.

### 2.4 Ethics

Ethical approval was granted by the Ethical–Scientific Committee of Universidad de Las Américas, under protocol CEC-FP-2023014. Participation was voluntary and anonymous. The consent form explained the study's purpose, confidentiality measures, and participants' rights to decline or withdraw at any time without repercussions.

### 2.5 Statistical analysis

Analyses were designed to (i) describe trichinellosis-related knowledge and preventive practices, (ii) quantify the association between knowledge and preventive practices while accounting for community and sociodemographic factors, and (iii) derive interpretable behavioral axes from multivariate item patterns to support intervention-oriented interpretation.

#### 2.5.1 Descriptive analysis

For each item, responses were summarized as counts and percentages of correct/expected responses for Knowledge and implemented behaviors for Practices, based on pre-specified dichotomization rules. Knowledge and Practices sum scores (0–9 each) were summarized using means and standard deviations, together with distributions.

#### 2.5.2 Regression modeling

Associations were assessed using generalized additive models (GAMs), which allow evaluation of potential non-linear relationships via penalized smooth terms while retaining standard regression interpretation when relationships are approximately linear. The preventive practices sum score was modeled as a function of the knowledge sum score, adjusting for community, sex, and age group. To evaluate potential non-linearity, a model including a penalized spline for Knowledge was compared against a purely linear specification; smoothing parameters were estimated using restricted maximum likelihood (REML). Because the spline did not meaningfully improve model fit, the linear specification was retained for parsimony and interpretability [22], [23]. For this knowledge–practice model, Gaussian errors with an identity link were used; the scaled-t family (mgcv *scat*) was reserved for the PCA-axis models to improve robustness to heavy-tailed residuals and potential outliers (Section 2.5.3). Although the practice score is bounded (0–9), Gaussian models with an identity link are commonly used for sum scores when residual diagnostics are adequate; diagnostics are provided in the Supplementary Data.

#### 2.5.3 Principal component analysis of item patterns

To summarize co-occurring response patterns across the 18 dichotomized items (9 Knowledge +9 Practices) and reduce dimensionality, principal component analysis (PCA) was conducted on the item matrix. Item columns were centered and scaled (z-scores) so that components reflected patterns of co-variation rather than differences in item variance. PCA was performed on participants with complete data for all 18 items. The proportion of variance explained by each component was recorded, and the first two components (PC1 and PC2) were retained a priori for interpretation and used as continuous trichinellosis-related behavioral axes.

To assess social patterning of the derived axes, PC1 and PC2 were modeled as outcomes with community, sex, and age group as predictors. To improve robustness to heavy-tailed residuals and potential outliers, these models were fitted using a scaled-t error distribution with an identity link (family = scat in mgcv).

Model adequacy was evaluated using residual diagnostics, Q–Q plots under the selected error distribution, and GAM diagnostic checks (including gam.check). When smooth terms were considered, basis-dimension diagnostics were reviewed. Statistical support was defined as *p* < 0.05. All analyses were conducted in R (via RStudio 2026.01.0 + 392) using the mgcv package [23], [24] and base R functions.

## 3 Results

### 3.1 Participant characteristics and item-level responses

A total of 180 adults from nine Mapuche communities in Contulmo (Arauco Province, Biobío Region, Chile) participated in the survey. The communities are designated as Community 1–9, with 20 participants per community. The sample comprised 97 men (53.9%) and 83 women (46.1%). Age groups were 18–39 years (*n* = 61; 33.9%), 40–59 years (*n* = 86; 47.8%), and 60 years or older (*n* = 33; 18.3%).

Item-level results using the dichotomized scoring method (correct/expected = 1; other responses = 0) are shown in Table 1, Table 2. Regarding knowledge, the average total score was 7.47 out of 9 (SD 1.21), corresponding to approximately 82.96% correct answers. The highest correct response rates were for identifying pork as a high-risk meat (K5: 96.7%) and recognizing undercooked pork as a transmission route (K3: 91.7%). Misconceptions remained about the “cooking” of meat with lemon juice (K7: 72.2% correct) and whether smoking kills *Trichinella* (K8: 76.7%). For practices, the mean score was 6.21 out of 9 (SD 2.13), indicating an average of 69.0% adherence to recommended practices. The most common practices included avoiding eating pork suspected of containing *Trichinella* (P6: 95.0%) and attending the local hospital for care (P9: 88.9%). Lower adherence was observed for weekly cleaning of feeding areas (P2: 57.8%) and for burying or disposing of infected meat (P7: 53.9%). Complete distribution data for multi-category responses are available in the Supplementary Data (Supplementary Tables S1–S2).

**Table 1:** Trichinellosis knowledge items (dichotomized) in Mapuche communities (*n* = 180).

Item	Knowledge item (correct response)	Correct n (%)
K1	Affects humans and animals	137 (76.1%)
K2	Cause is a parasitic worm in meat	129 (71.7%)
K3	Infection via eating raw/undercooked pork	165 (91.7%)
K4	Typical symptoms (diarrhoea and muscle/joint pain)	160 (88.9%)
K5	High-risk meat: pork	174 (96.7%)
K6	Pigs infected by eating rodents	155 (86.1%)
K7	Lemon juice does not “cook” meat	130 (72.2%)
K8	Smoking does not kill *Trichinella*	138 (76.7%)
K9	Risk if pork is eaten without veterinary testing/inspection	156 (86.7%)

*Note:* Values are the number (%) coded as correct (=1) according to pre-specified scoring rules.

**Table 2:** Trichinellosis preventive practice items (dichotomized) in Mapuche communities (*n* = 180).

Item	Practice item (expected preventive behaviour)	Expected n (%)
P1	Eats pork well cooked	151 (83.9%)
P2	Cleans pig feeding area weekly	104 (57.8%)
P3	Tests home-slaughtered pork with a veterinarian before consumption	118 (65.6%)
P4	Does not use lemon juice to check pork for *Trichinella*	117 (65.0%)
P5	Does not smoke pork to eliminate *Trichinella*	137 (76.1%)
P6	Does not eat pork known to contain *Trichinella*	171 (95.0%)
P7	Disposes/buries pork known to contain *Trichinella*	97 (53.9%)
P8	Does not rely on Machi alone for trichinellosis treatment	63 (35.0%)
P9	Seeks care at the local hospital when trichinellosis is suspected	160 (88.9%)

*Note:* Values are the number (%) coded as expected preventive behaviour (=1) according to pre-specified scoring rules.

### 3.2 Association between knowledge and preventive practices

**Fig. 1:**
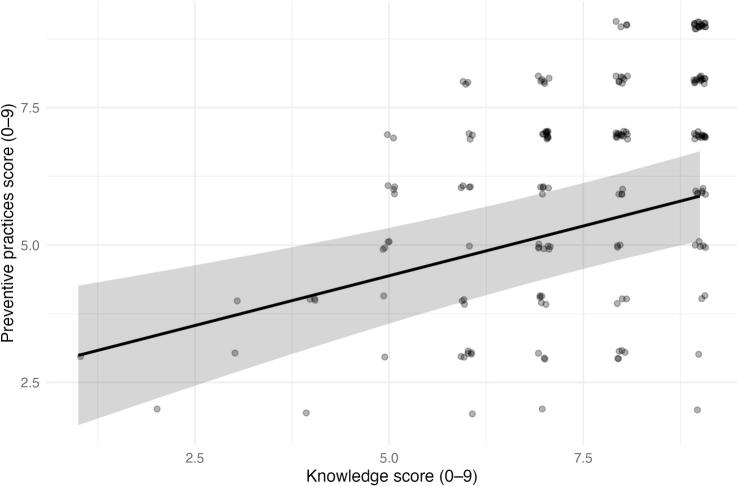
Adjusted relationship between knowledge and preventive practices. Model-predicted preventive practices score (0–9) across the observed range of the knowledge score (0–9), holding covariates at their reference levels (Community 1, Female, age 18–39). The solid line shows the adjusted mean prediction and the shaded band the 95% confidence interval; points show observed values for context.

Preventive practices (sum-score, 0–9) increased with higher trichinellosis knowledge (sum-score, 0–9) in adjusted analyses (Table 3; Fig. 1). After controlling for community, sex, and age group, each additional correct knowledge item was associated with an average increase of 0.36 points in the preventive practices score (β = 0.362; 95% CI 0.208 to 0.516; *p* < 0.001). In practical terms, respondents with more accurate knowledge tended to report more recommended preventive behaviors.

**Table 3:** Adjusted association between knowledge and preventive practices (sum-score model; *n* = 180).

Term	β	SE	z	*p*-value	95% CI
Intercept	2.631	0.710	3.706	<0.001	1.239–4.022
Knowledge score (0–9)	0.362	0.078	4.618	<0.001	0.208–0.516
Community 2	1.234	0.467	2.639	0.008	0.318–2.150
Community 3	0.525	0.468	1.122	0.262	−0.392 - 1.443
Community 4	2.032	0.479	4.245	<0.001	1.094–2.971
Community 5	0.839	0.482	1.739	0.082	−0.106 - 1.785
Community 6	0.639	0.467	1.368	0.171	−0.277 - 1.556
Community 7	3.078	0.483	6.368	<0.001	2.131–4.025
Community 8	0.499	0.472	1.057	0.291	−0.426 - 1.425
Community 9	−0.301	0.476	−0.633	0.527	−1.234 - 0.632
Sex: Male	−0.019	0.228	−0.085	0.932	−0.466 - 0.427
Age group: 40–59	−0.148	0.267	−0.557	0.578	−0.671 - 0.374
Age group: 60+	0.048	0.346	0.140	0.889	−0.629 - 0.726

Outcome: preventive practices sum-score (0–9). Predictor: knowledge sum-score (0–9). Model adjusted for community, sex, and age group.

*Note:* Coefficients for communities are contrasts versus **Community 1**; sex is contrasted versus **Female**; age groups are contrasted versus **18–39**. β represents the adjusted mean difference in the preventive practices sum-score (0–9).

Adjusted practice scores also differed across communities (Table 3). Compared with Community 1 (reference), Communities 2, 4, and 7 had higher preventive practice scores after adjustment (all *p* < 0.01), whereas the remaining community contrasts were not statistically significant. Sex and age group were not associated with preventive practices in this model (*p* > 0.05 for all).

Non-linearity of the knowledge–practice relationship was evaluated by comparing a linear term versus a penalized spline for knowledge; the spline did not improve model fit meaningfully, so the linear specification was retained for parsimony (Fig. 1 shows the resulting monotonic increase).

### 3.3 Principal component analysis

**Fig. 2:**
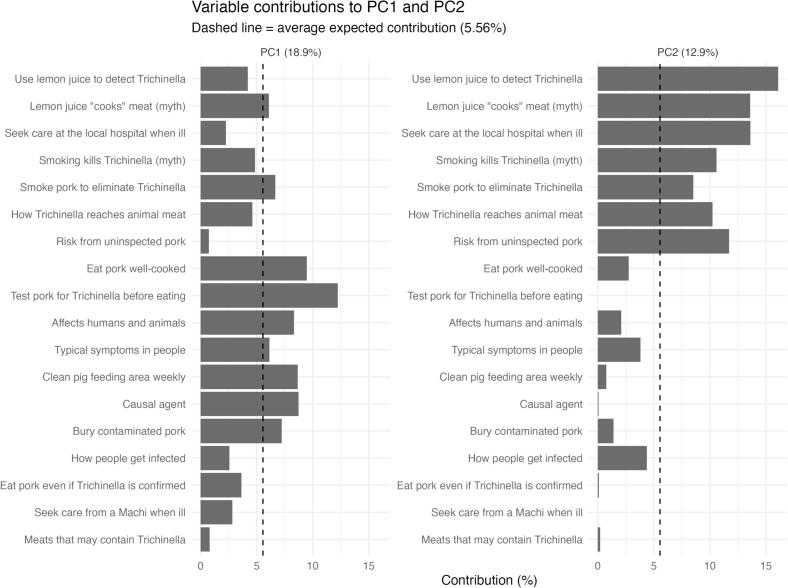
Variable contributions to the first two principal components (PC1 and PC2). Bars show the percentage contribution (based on squared loadings) of each dichotomized item to PC1 (left) and PC2 (right). The dashed vertical line indicates the average expected contribution if all items contributed equally (5.56%). Items with contributions above this line most strongly define each axis.

Principal component analysis (PCA) of the 18 dichotomized items identified two main behavioral dimensions (PC1 and PC2), explaining 18.9% and 12.9% of the variance, respectively (cumulative 31.8%). PC1 was defined by core prevention behaviors and knowledge items (e.g., testing pork for *Trichinella* before consumption, eating pork well-cooked, cleaning the pig feeding area, and basic etiologic/transmission knowledge) and was interpreted as the Trichinellosis Prevention Axis (TPA) (Fig. 2; Supplementary Fig. S2). PC2 was primarily defined by items reflecting food-preparation misconceptions, particularly beliefs and practices involving lemon juice and smoking (Fig. 2; Supplementary Fig. S2). For interpretability, PC2 was oriented so that higher values indicate greater endorsement of food-preparation misconceptions (i.e., a lower probability of rejecting these myths) and was labeled the Food-Preparation Misconceptions Axis (FPMA).

Because contributions are based on squared loadings, this figure indicates *importance* but not the *direction* (sign) of the relationship; axis orientation is therefore clarified using the PCA loading map (Supplementary Fig. S2). PC1 was interpreted as the Trichinellosis Prevention Axis (TPA). PC2 was interpreted as the Food-Preparation Misconceptions Axis (FPMA) and oriented so that higher values indicate greater endorsement of food-preparation misconceptions.

### 3.4 Differences in PCA axes across communities and demographic groups

#### 3.4.1 Trichinellosis Prevention Axis (TPA; PC1)

**Fig. 3:**
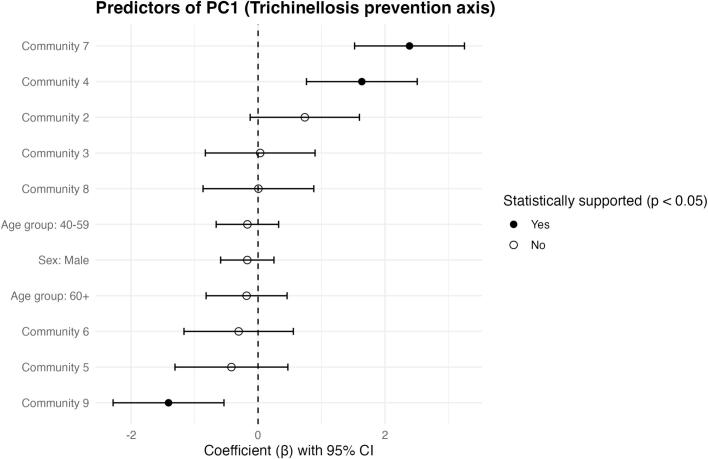
Predictors of the Trichinellosis Prevention Axis (TPA; PC1). Points show adjusted regression coefficients (β) with 95% confidence intervals from a robust scaled-t identity-link model for PC1. Coefficients represent contrasts relative to the reference community (Community 1), with sex referenced to female and age group referenced to 18–39 years. The vertical dashed line indicates the null (β = 0). Filled points denote effects with *p* < 0.05. Positive coefficients indicate higher TPA values (i.e., stronger alignment with core prevention behaviors/knowledge captured by PC1) after adjustment for sex and age group.

After adjustment for sex and age group, TPA scores differed across communities (Fig. 3). Compared with the reference community, higher TPA scores were observed in Communities 4 and 7, whereas Community 9 showed lower TPA scores; the remaining communities did not differ significantly from the reference at the 0.05 level. In the same adjusted model, neither sex nor age group was associated with TPA (Fig. 3).

#### 3.4.2 Food-Preparation Misconceptions Axis (FPMA; PC2)

**Fig. 4:**
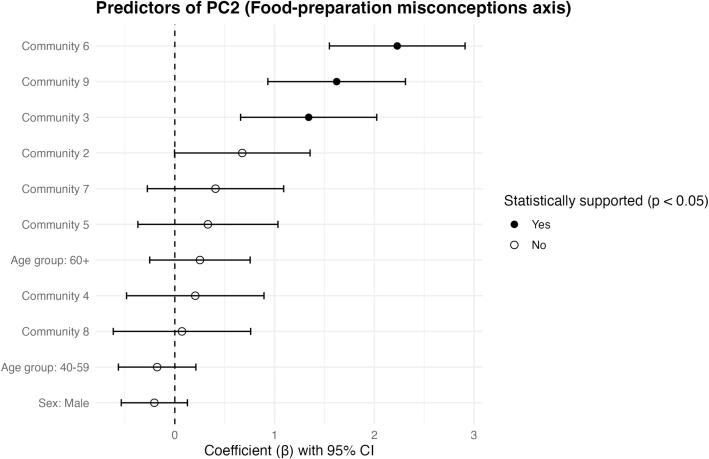
Predictors of the Food-Preparation Misconceptions Axis (FPMA; PC2). Points show adjusted regression coefficients (β) with 95% confidence intervals from a robust scaled-t identity-link model for PC2. Coefficients represent contrasts relative to the reference community (Community 1), with sex referenced to female and age group referenced to 18–39 years. The vertical dashed line indicates the null (β = 0). Filled points denote effects with *p* < 0.05. PC2 was oriented for interpretability so that higher FPMA values indicate greater endorsement of food-preparation misconceptions (e.g., lemon juice and smoking-related myths), after adjustment for sex and age group.

After adjustment for sex and age group, the FPMA scores also differed across communities (Fig. 4). Compared with the reference community, higher FPMA scores (i.e., greater endorsement of food-preparation misconceptions) were observed in Communities 3, 6, and 9; no other communities showed statistically significant differences at the 0.05 level. Sex and age group were not statistically supported predictors of FPMA in the adjusted regression model (Fig. 4).

Marginal adjusted means of FPMA by sex and age group are shown in Supplementary Figs. S3–S4. While these plots suggest directional patterns (e.g., slightly higher FPMA values among women and among participants aged ≥60 years), the associated uncertainty intervals overlap substantially, and these patterns should be interpreted as descriptive trends rather than evidence of group differences.

In summary, preventive practices increased with higher knowledge, and multivariate item patterns condensed into two meaningful One Health–relevant axes: a Trichinellosis Prevention Axis (TPA) and a Food-Preparation Misconceptions Axis (FPMA). Adjusted models indicated that differences across communities persisted after controlling for sex and age group, underscoring that trichinellosis risk-related behaviors are socially and territorially patterned rather than homogeneous within the study area.

## 4 Discussion

This cross-sectional survey of adults from nine Mapuche communities in Contulmo provides three main findings with direct relevance for One Health prevention of trichinellosis. First, participants showed high overall knowledge (mean 7.47/9), but specific misconceptions persisted—most notably beliefs related to lemon juice and smoking as “controls” for *Trichinella*. Second, several preventive practices showed lower adherence, particularly those that require routine animal management and safe disposal actions (e.g., weekly cleaning of pig feeding areas and disposal/burial of infected meat). Third, knowledge was positively associated with preventive practices in adjusted analyses, yet meaningful between-community differences remained after controlling for sex and age group, indicating territorial patterning of behaviors and beliefs rather than homogeneity across the study area.

The combination of high knowledge and persistent food-preparation myths is epidemiologically important because these misconceptions target the final step in risk reduction: decisions made at the point of preparation and consumption [4]. In contexts where home slaughter and household preparation are common, practical “rules of thumb” (e.g., acid, smoke, or other sensory cues as indicators of safety) can endure even when biomedical knowledge is generally high. In this study, the items involving lemon juice and smoking remained among the least consistently endorsed, suggesting that trichinellosis prevention strategies must address not only “what *Trichinella* is,” but also how safety is judged in everyday preparation routines and how these judgments are reinforced within households and communities. Importantly, trichinellosis remains strongly linked to consumption of raw or undercooked meat, reinforcing that safety judgments based on preparation style or sensory cues should not substitute for testing and adequate cooking [3], [25].

Similar One Health work in Indigenous contexts illustrates how structural constraints—such as remoteness and limited access to sanitation and veterinary services—can sustain zoonotic risk and constrain prevention options [[13], [15]]. The observed practice gaps in our study correspond to points at which the animal–environment interface can sustain transmission when prevention is difficult to implement [26]. Cleaning feeding areas and safely disposing of potentially infected pork are not merely “behaviors”; they depend on feasible routines, material conditions, and access to appropriate disposal options. Likewise, testing home-slaughtered pork requires service availability, transport, and a practicable pathway for sample submission [27]. Therefore, although the adjusted association between knowledge and practices indicates that knowledge is meaningfully related to safer self-reported behaviors, the remaining gaps suggest that information alone may be insufficient when prevention depends on household capacity and local infrastructure.

The PCA provided a useful synthesis of how knowledge and practices co-occur. The Trichinellosis Prevention Axis (TPA) grouped core preventive behaviors (testing prior to consumption, thorough cooking, routine hygiene) with aligned etiologic/transmission knowledge, consistent with a prevention “bundle” likely constrained by household routines and service access. The Food-Preparation Misconceptions Axis (FPMA) captured a more specific cluster of preparation myths (lemon juice and smoking). Notably, community-level differences were statistically significant on both axes after adjustment for sex and age group, supporting the conclusion that prevention-relevant patterns are territorially structured. At the same time, sex and age group were not statistically supported predictors in the adjusted FPMA model; therefore, any apparent separation in adjusted means plots should be treated as descriptive trends rather than evidence of demographic effects in this dataset.

A stronger comparison to global Indigenous One Health literature further supports the interpretation that culturally embedded food practices and structural feasibility are central determinants of zoonotic risk [28]. In Arctic Indigenous contexts [29], trichinellosis prevention has required approaches that move beyond generic risk messaging to strategies that respect food traditions while strengthening practical protection—most notably through community-based testing programs and locally grounded communication [30]. More broadly, syntheses of One Health research in Indigenous communities [13] emphasize that effective interventions are typically community-led, culturally safe, and designed around local priorities and constraints (including service access, infrastructure, and the lived realities of food systems), rather than framed as simple “knowledge deficits” [13], [14], [15], [30]. These observations align with the present finding that community differences persisted after demographic adjustment in both the prevention and misconception dimensions.

Finally, the results provide a concrete basis for intercultural collaboration, linked to specific survey signals rather than described abstractly. Two items illustrate this clearly: high reported care-seeking at the local hospital (P9) alongside low endorsement of “not relying on Machi alone” (P8). This pattern is consistent with medical pluralism—coexisting use of biomedical and traditional health systems—rather than disengagement from formal care. In practical terms, prevention initiatives are likely to be more credible and adoptable if they integrate intercultural health actors and community leadership, and if they frame myth-correction and prevention behaviors (testing, safe disposal, hygienic routines) in culturally safe ways that do not undermine trusted local knowledge structures or exclude locally legitimate care pathways. Evidence from other Indigenous settings underscores that uptake of prevention is strongly shaped by trust and by who delivers the message; community leaders and Elders can be pivotal partners for culturally credible risk communication and practice change [31].

Behavioral heterogeneity often limits the effectiveness of generic risk messaging; identifying co-occurring behavior patterns can support more targeted [32], context-specific intervention design rather than one-size-fits-all approaches [33]. Taken together, the findings suggest that trichinellosis prevention in this setting is best approached as a territorially tailored One Health implementation challenge: strengthen the feasibility of the TPA “bundle” in communities with lower prevention alignment, and address the FPMA cluster through targeted, culturally grounded messaging that directly engages the lemon-juice/smoking myths. This evidence-based targeting—grounded in adjusted models and interpretable multivariate structure—provides a practical foundation for designing and evaluating community-specific interventions.

## 5 Limitations

Several limitations should be considered when interpreting these findings. First, participants were recruited through convenience sampling in nine Mapuche communities; therefore, the sample may not be representative of all Mapuche communities in Contulmo or of other Indigenous populations in Chile, and external generalizability is limited. Second, survey responses reflect self-reported knowledge and practices, which are subject to recall error and social desirability bias [34], potentially inflating endorsement of recommended preventive behaviors or under-reporting socially undesirable practices.

Third, the study did not include direct observation of animal husbandry, slaughter, meat handling, or carcass disposal behaviors; consequently, reported practices cannot be validated against observed behaviors. Fourth, the One Health interpretation in this study should be understood as a conceptual integration of human, animal, and environmental prevention points based on reported KAP patterns, rather than as an empirical test of transmission pathways. Because we did not collect parallel veterinary and environmental measurements (e.g., pig infection status, household pig-rearing conditions, rodent abundance), we cannot quantify the relative contribution of specific human–animal–environment pathways, validate whether reported practices track measurable exposure risk, or explain community differences through observed ecological or service-access determinants. Finally, the cross-sectional design precludes causal inference: observed associations (e.g., between knowledge and preventive practices) should be interpreted as correlational patterns rather than as evidence that increasing knowledge alone would necessarily produce behavioral change, without accounting for feasibility and context-specific determinants.

## 6 Conclusions

This study reveals that adults from nine Mapuche communities in Contulmo have high levels of knowledge about trichinellosis, but adherence to various preventive practices remains low—especially regarding routine animal management, access to veterinary testing, and proper disposal of infected meat. While knowledge was positively associated with preventive practices, even after adjustment for community, sex, and age, persistent differences across communities underscore the need to consider territorial feasibility and local capacity for implementation. PCA grouped responses into two key dimensions: a Trichinellosis Prevention Axis reflecting core preventative behaviors and related knowledge, and a Food-Preparation Misconceptions Axis highlighting persistent myths about lemon juice and smoking.

Together, these findings frame trichinellosis prevention as a One Health challenge that requires culturally safe communication alongside practical, community-specific enabling conditions – such as making testing and safe disposal options more accessible - to translate knowledge into sustained practice.

## CRediT authorship contribution statement

**Tania Grant-Riquelme:** Writing – review & editing, Writing – original draft, Visualization, Validation, Supervision, Resources, Project administration, Methodology, Investigation, Funding acquisition, Formal analysis, Data curation, Conceptualization. **Yanina Poblete:** Writing – review & editing, Visualization, Validation, Software, Methodology, Formal analysis, Data curation. **Marcela Fresno:** Writing – original draft. **Cecilia Baumberger:** Writing – original draft, Methodology, Formal analysis. **Italo Fernandez Fonseca:** Writing – original draft. **Christopher Hamilton-West:** Writing – review & editing. **Francisca Di Pillo:** Writing – review & editing, Writing – original draft, Visualization, Validation, Supervision, Software, Resources, Project administration, Methodology, Investigation, Funding acquisition, Formal analysis, Data curation, Conceptualization.

## Funding

This project was self-funded and did not receive any specific grants from funding agencies.

## Declaration of competing interest

The authors declare no conflict of interest.

## Data Availability

Data will be made available on request.

## Appendix A Supplementary data

Supplementary material Image 1
