# Supplementary material for: Trichinellosis knowledge and preventive practices in Mapuche communities of southern Chile: Evidence for targeted One Health implementation
Source: One Health. 2026 Feb 17;22:101366. doi: 10.1016/j.onehlt.2026.101366 (PMC12962113; doi:10.1016/j.onehlt.2026.101366)
Supplement: Supplementary file 1 — Supplementary material [file mmc1.docx]

**Supplementary Table 1.** Knowledge related to trichinellosis among Mapuche communities in Contulmo

| **Question Nº** | **Knowledge Questions** | **Answers** | **n** | **%** |
| --- | --- | --- | --- | --- |
| 1 | Does trichinellosis affect both humans and animals? | Yes | 137 | 76.11 |
|  |  | No | 5 | 2.78 |
|  |  | Only humans | 26 | 14.44 |
|  |  | I don't know | 12 | 6.67 |
| 2 | What is the cause of trichinellosis? | A parasitic worm in meat | 129 | 71.67 |
|  |  | Flies | 38 | 21.11 |
|  |  | Something in the water | 2 | 1.11 |
|  |  | I don't know | 11 | 6.11 |
| 3 | How can people become infected? | Eating raw or undercooked pork | 165 | 91.67 |
|  |  | Incorrect hand washing | 2 | 1.11 |
|  |  | Other person-to-person transmission | 0 | 0 |
|  |  | I don't know | 13 | 7.22 |
| 4 | What are the symptoms of trichinellosis in humans? | Diarrhoea and bone pain | 160 | 88.89 |
|  |  | Itching | 3 | 1.67 |
|  |  | I don't know | 17 | 9.44 |
| 5 | In what kind of meat can trichinellosis be present? | Pigs | 174 | 96.67 |
|  |  | Cows | 3 | 1.67 |
|  |  | Horse | 0 | 0 |
|  |  | I don't know | 3 | 1.67 |
| 6 | How can pigs get trichinellosis? | Eating rodents | 155 | 86.11 |
|  |  | Drinking contaminated water | 6 | 3.33 |
|  |  | Contact with animal faeces | 7 | 3.89 |
|  |  | I don't know | 12 | 6.67 |
| 7 | Can lemon juice be used to cook meat? | No | 130 | 72.22 |
|  |  | Yes | 14 | 7.78 |
|  |  | Sometimes | 14 | 12.22 |
|  |  | I don't know | 22 | 7.78 |
| 8 | Does smoking eliminate Trichinella? | No | 138 | 76.67 |
|  |  | Yes | 16 | 8.89 |
|  |  | Sometimes | 5 | 2.78 |
|  |  | I don't know | 21 | 11.67 |
| 9 | Does consuming meat without veterinary inspection constitute a risk? | Yes | 156 | 86.67 |
|  |  | No | 4 | 2.22 |
|  |  | Sometimes | 16 | 8.89 |
|  |  | I don't know | 4 | 2.22 |

**Supplementary Table 2**. Practices related to trichinellosis among Mapuche communities in Contulmo

| **Question Nº** | **Practice Questions** | **Answers** | **n** | **%** |
| --- | --- | --- | --- | --- |
| 1 | How often do you eat pork fully cooked? | Always | 151 | 83.89 |
|  |  | Almost always | 25 | 13.89 |
|  |  | I don't know | 0 | 0.00 |
|  |  | Rarely | 0 | 0.00 |
|  |  | Never | 4 | 2.22 |
| 2 | I clean the animals' feeders once a week | Always | 104 | 57.78 |
|  |  | Almost always | 59 | 32.78 |
|  |  | I don't know | 4 | 2.22 |
|  |  | Rarely | 5 | 2.78 |
|  |  | Never | 8 | 4.44 |
| 3 | I get the meat examined by a veterinarian after slaughtering the pig | Always | 118 | 65.56 |
|  |  | Almost always | 36 | 20.00 |
|  |  | I don't know | 3 | 1.67 |
|  |  | Rarely | 13 | 7.22 |
|  |  | Never | 10 | 5.56 |
| 4 | I use lemon juice to check if the meat contains Trichinella | Always | 13 | 7.22 |
|  |  | Almost always | 7 | 3.89 |
|  |  | I don't know | 6 | 3.33 |
|  |  | Rarely | 37 | 20.56 |
|  |  | Never | 117 | 65.00 |
| 5 | I smoke the meat to eliminate Trichinella | Always | 5 | 2.78 |
|  |  | Almost always | 3 | 1.67 |
|  |  | I don't know | 3 | 1.67 |
|  |  | Rarely | 32 | 17.78 |
|  |  | Never | 137 | 76.11 |
| 6 | I eat the meat even when it's known to contain Trichinella | Always | 3 | 1.67 |
|  |  | Almost always | 2 | 1.11 |
|  |  | I don't know | 1 | 0.56 |
|  |  | Rarely | 3 | 1.67 |
|  |  | Never | 171 | 95.00 |
| 7 | I bury the meat when it has Trichinella | Always | 97 | 53.89 |
|  |  | Almost always | 10 | 5.56 |
|  |  | I don't know | 15 | 8.33 |
|  |  | Rarely | 32 | 17.78 |
|  |  | Never | 26 | 14.44 |
| 8 | When someone falls ill with trichinellosis, I consult the Machi (healer) | Always | 19 | 10.56 |
|  |  | Almost always | 35 | 19.44 |
|  |  | I don't know | 42 | 23.33 |
|  |  | Rarely | 21 | 11.67 |
|  |  | Never | 63 | 35.00 |
| 9 | When someone falls ill with trichinellosis, I take him/her to the hospital in town | Always | 160 | 88.89 |
|  |  | Almost always | 12 | 6.67 |
|  |  | I don't know | 3 | 1.67 |
|  |  | Rarely | 3 | 1.67 |
|  |  | Never | 2 | 1.11 |

**
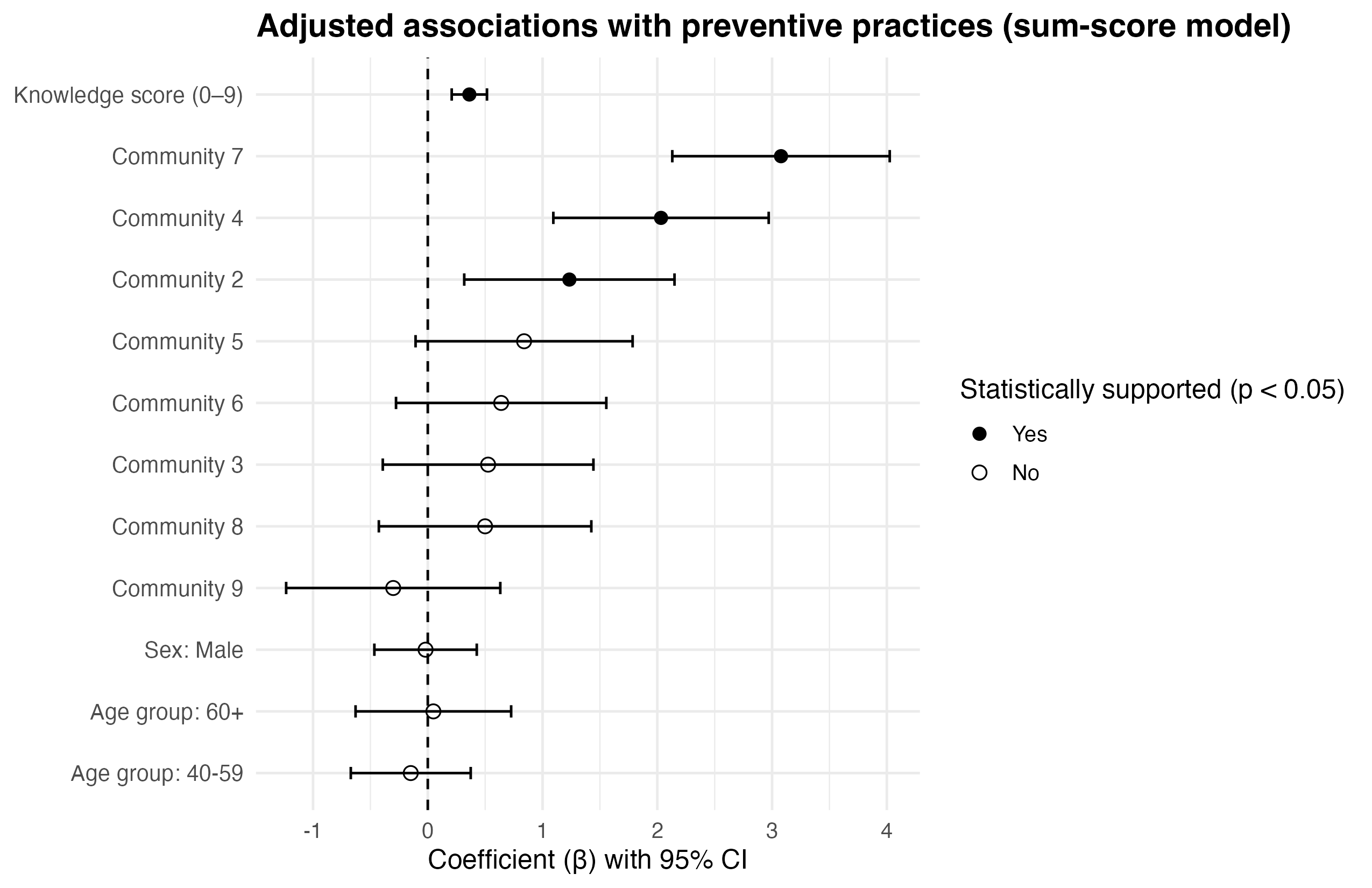
**

**Supplementary Figure S1. Adjusted associations with preventive practices (sum-score model).** Points show regression coefficients (β) and horizontal bars represent 95% confidence intervals from the scaled-t model predicting preventive practices (0–9) as a function of knowledge (0–9), community (reference: Community 1), sex (reference: Female), and age group (reference: 18–39 years). Filled points indicate p < 0.05; the vertical dashed line marks β = 0.


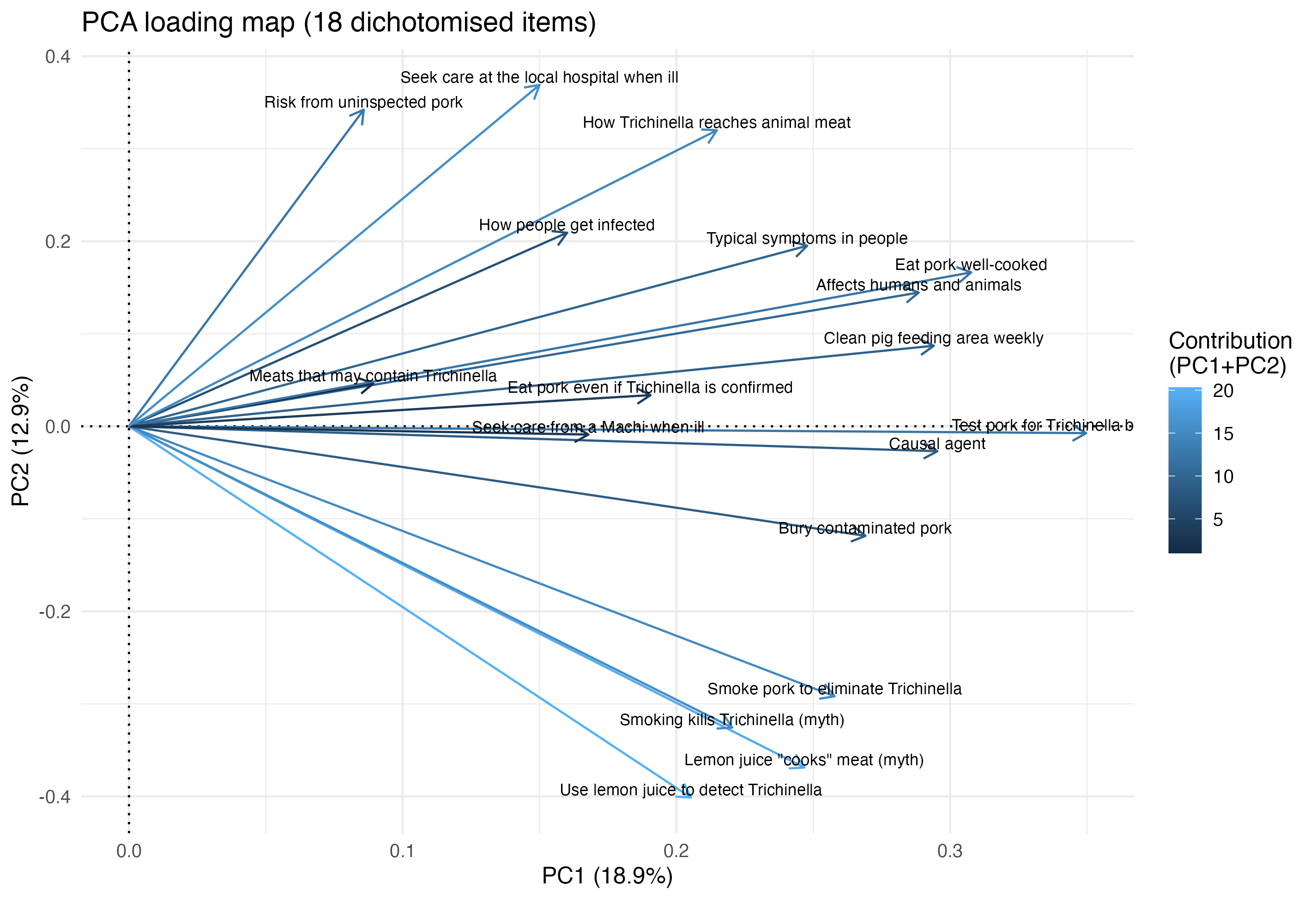


**Supplementary Figure S2. PCA loading map for the 18 dichotomized items (PC1–PC2). Arrows represent item loadings on the first two principal components (PC1 and PC2). Arrow direction indicates the sign of the loading (items pointing in the same direction are positively correlated; opposite directions indicate negative correlation), and arrow length reflects the strength of association with the component space. The percentage of variance explained by each component is shown on the axes. This loading map supports interpretation of axis direction (sign), which is not conveyed by the contribution plot (Figure 4), where contributions are based on squared loadings and therefore have no sign. PC2 is oriented so that higher values indicate greater endorsement of food-preparation misconceptions (FPMA).**

**Supplementary Table 3. Scaled-t regression results for PC1 (Trichinellosis Prevention Axis; TPA) (n = 180)**

Reference levels: Community 1, Female, Age group 18–39. Positive β indicates a higher TPA score (more alignment with prevention behaviors/knowledge) vs the reference.

| **Term** | **β** | **SE** | **z** | **p** | **95% CI (lower)** | **95% CI (upper)** |
| --- | --- | --- | --- | --- | --- | --- |
| Intercept | 0.069 | 0.376 | 0.18 | 0.854 | -0.667 | 0.805 |
| Community 2 | 0.737 | 0.439 | 1.68 | 0.094 | -0.124 | 1.598 |
| Community 3 | 0.034 | 0.441 | 0.08 | 0.939 | -0.831 | 0.899 |
| Community 4 | 1.636 | 0.445 | 3.67 | <0.001 | 0.763 | 2.509 |
| Community 5 | -0.42 | 0.454 | -0.92 | 0.355 | -1.309 | 0.47 |
| Community 6 | -0.306 | 0.439 | -0.7 | 0.485 | -1.167 | 0.555 |
| Community 7 | 2.416 | 0.468 | 5.16 | <0.001 | 1.498 | 3.333 |
| Community 8 | -0.235 | 0.448 | -0.52 | 0.601 | -1.113 | 0.644 |
| Community 9 | -1.135 | 0.445 | -2.55 | 0.011 | -2.007 | -0.262 |
| Sex: Male | -0.103 | 0.22 | -0.47 | 0.64 | -0.534 | 0.328 |
| Age group: 40-59 | -0.138 | 0.259 | -0.53 | 0.594 | -0.646 | 0.37 |
| Age group: 60+ | -0.154 | 0.335 | -0.46 | 0.646 | -0.81 | 0.502 |

**Supplementary Table 4. Scaled-t regression results for PC2 (Food-Preparation Misconceptions Axis; FPMA) (n = 180)**

Reference levels: Community 1, Female, Age group 18–39. FPMA is oriented so that higher β indicates more misconceptions vs the reference.

| Term | β | SE | z | p | 95% CI (lower) | 95% CI (upper) |
| --- | --- | --- | --- | --- | --- | --- |
| Intercept | -0.612 | 0.296 | -2.07 | 0.039 | -1.193 | -0.031 |
| Community 2 | 0.676 | 0.347 | 1.95 | 0.051 | -0.003 | 1.356 |
| Community 3 | 1.342 | 0.348 | 3.85 | <0.001 | 0.659 | 2.025 |
| Community 4 | 0.205 | 0.352 | 0.58 | 0.56 | -0.484 | 0.894 |
| Community 5 | 0.332 | 0.358 | 0.93 | 0.354 | -0.37 | 1.034 |
| Community 6 | 2.251 | 0.456 | 4.94 | <0.001 | 1.358 | 3.144 |
| Community 7 | -0.196 | 0.348 | -0.56 | 0.573 | -0.878 | 0.486 |
| Community 8 | 0.055 | 0.352 | 0.16 | 0.876 | -0.634 | 0.744 |
| Community 9 | 1.496 | 0.479 | 3.12 | 0.002 | 0.556 | 2.437 |
| Sex: Male | -0.319 | 0.213 | -1.5 | 0.134 | -0.737 | 0.098 |
| Age group: 40-59 | -0.28 | 0.177 | -1.58 | 0.115 | -0.628 | 0.068 |
| Age group: 60+ | 0.215 | 0.179 | 1.2 | 0.227 | -0.136 | 0.565 |


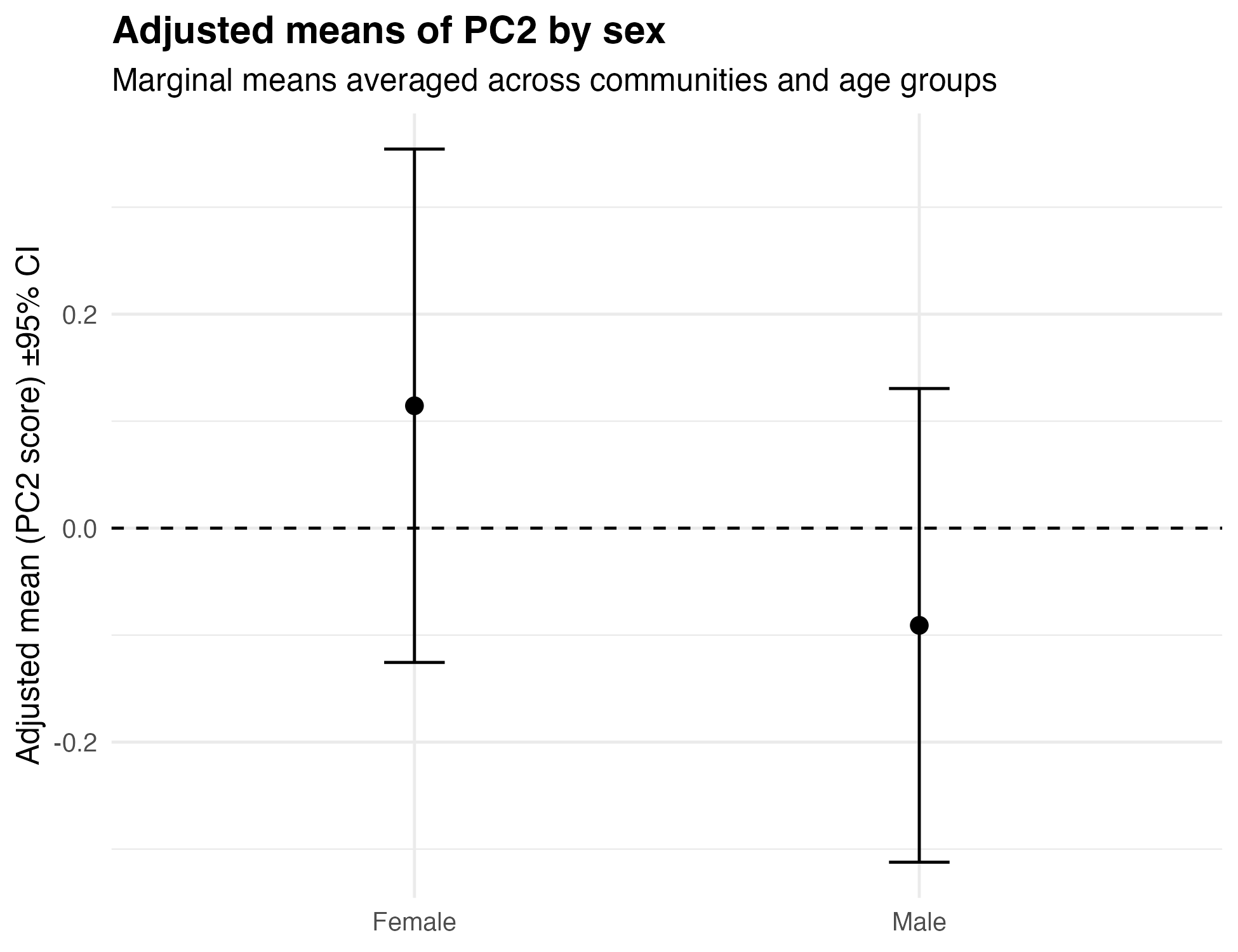


**Supplementary Figure S3. Adjusted marginal means of FPMA (PC2) by sex.**
Points show model-based adjusted means of PC2 with 95% confidence intervals, averaged across communities and age groups (marginal standardization). The horizontal dashed line marks zero on the PC2 scale. Higher values indicate greater endorsement of food-preparation misconceptions (FPMA orientation).


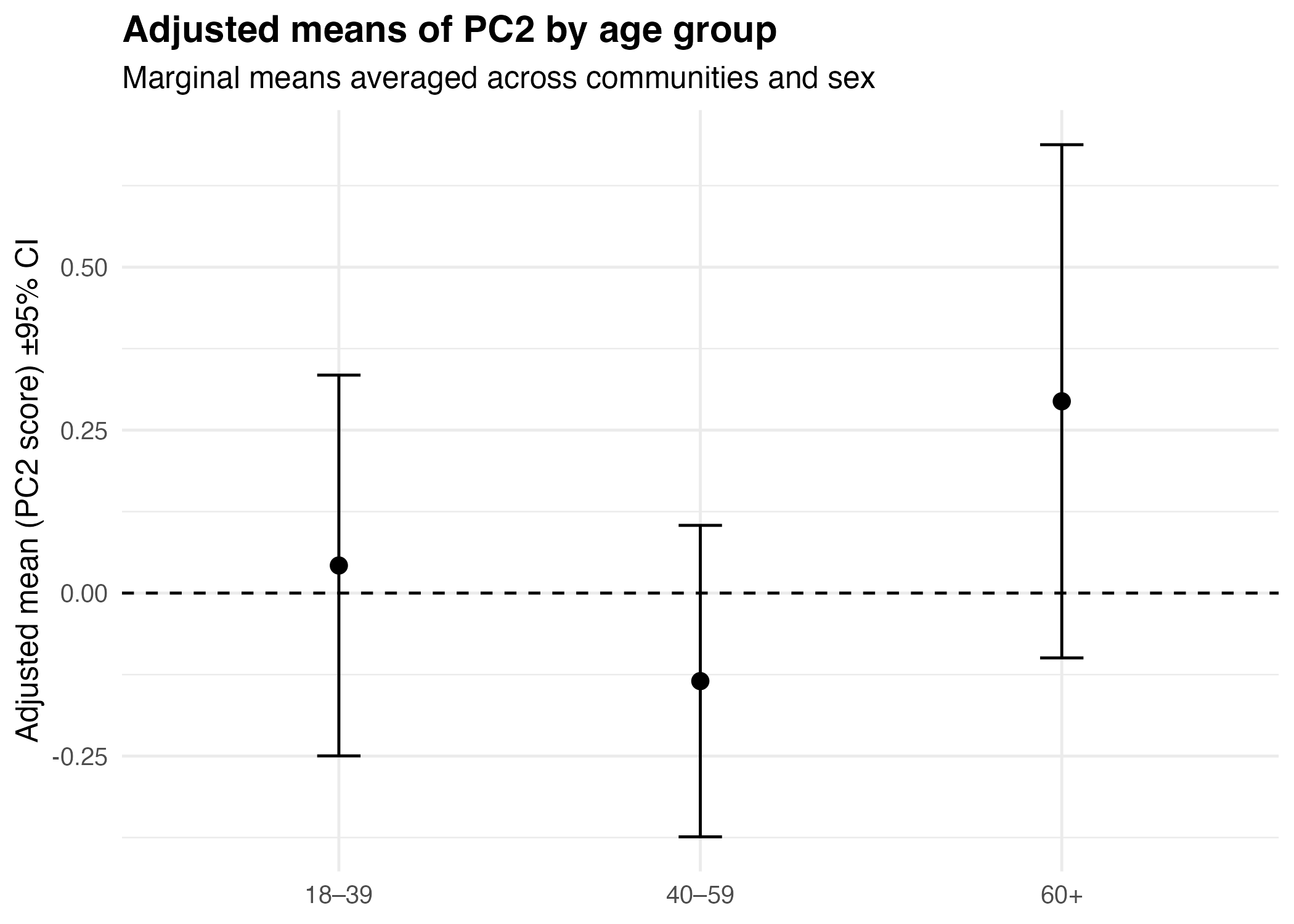


**Supplementary Figure S4. Adjusted marginal means of FPMA (PC2) by age group.**
Points show model-based adjusted means of PC2 with 95% confidence intervals, averaged across communities and sex (marginal standardization). The horizontal dashed line marks zero on the PC2 scale. Higher values indicate greater endorsement of food-preparation misconceptions (FPMA orientation).
